# Smoking Cessation Services by Community Pharmacists: Real-World Practice in Thailand

**DOI:** 10.3390/ijerph182211890

**Published:** 2021-11-12

**Authors:** Sunee Lertsinudom, Pentipa Kaewketthong, Tamonwan Chankaew, Dujrudee Chinwong, Surarong Chinwong

**Affiliations:** 1 Division of Clinical Pharmacy, Faculty of Pharmaceutical Sciences, Khon Kaen University, Khon Kaen 40002, Thailand; lsunee@kku.ac.th; 2 Smoke Free Pharmacy Network, Community Pharmacy Foundation, Bangkok 10110, Thailand; pkaewketthong@gmail.com; 3 Research and Training Center for Enhancing Quality of Life of Working-Age People, Faculty of Nursing, Khon Kaen University, Khon Kaen 40002, Thailand; 4 Pharmacy Department, Rajavithi Hospital, Bangkok 10400, Thailand; tamonwanchankaew@gmail.com; 5 Department of Pharmaceutical Care, Faculty of Pharmacy, Chiang Mai University, Chiang Mai 50200, Thailand; dujrudee.c@cmu.ac.th; 6 Cluster of Excellence on Biodiversity-Based Economic and Society (B.BES-CMU), Chiang Mai University, Chiang Mai 50200, Thailand

**Keywords:** Thailand, smoking cessation, community pharmacy, real-world practice

## Abstract

This study aimed to report the effectiveness of community pharmacy smoking cessation services in 13 health regions in Thailand using a retrospective data-collecting method from the Foundation of Community Pharmacy database. The participants were smokers aged at least 18 years. The outcomes were the abstinence of smoking at least 30 consecutive days by self-report only and self-report with exhaled CO level <7 ppm (if available), the number of cigarettes smoked daily, exhaled carbon monoxide (exhaled CO), and % peak expiratory flow rate (%PEFR); smokers measured these outcomes before and after receiving the smoking cessation services. Of 58 community pharmacies, 532 smokers (93% male, mean age of 42.4 ± 14.9 years) received smoking cessation services from community pharmacists. Of 235 smokers with complete data, 153 (28.8%, 153/532) smokers reported smoking abstinence by self-report. The mean number of cigarettes smoked daily reduced from 15.3 ± 8.7 to 1.9 ± 3.8 cigarettes, *p*-value < 0.001. The exhaled CO levels of smokers significantly reduced from 11.7 ± 5.9 ppm to 7.2 ± 4.4 ppm, *p*-value < 0.001. The %PEFR also significantly increased from 84.2 ± 19.4 to 89.5 ± 19.5, *p*-value < 0.001. In conclusion, Thai community pharmacy smoking cessation services could aid smokers to quit smoking. This study is the outcome of the real-world community pharmacy smoking cessation service; policymakers should consider this service to be included in the national healthcare policy.

## 1. Introduction

Smoking is one of the main leading causes of illness, disability, and death globally. Over 8 million deaths have been due to tobacco use, either by direct smoking or second-hand smoke [1]. In the U.S., almost 40 million adults are active smokers, and almost one half of smokers die prematurely due to smoking. The medical costs or any smoking-related disease cost totals over $170 billion yearly [2]. Tobacco use is one of the International Classifications of Disease (ICD 10), diagnosis code Z72.0 [3]. It has become a global noncommunicable disease (NCD) target to reduce tobacco use because it constitutes one of the risk factors leading to much acute or chronic illness, including chronic obstructive pulmonary disease (COPD), lung cancer, or coronary artery disease (CAD) [4]. Due to this harm, the World Health Organization (WHO) promoted healthcare facilities to encourage smokers to use smoking cessation services to reduce the risk of developing related illnesses and ultimately prevent death [5].

According to the WHO report on the global tobacco epidemic, 2019, Country Report: Thailand [5], the latest survey completed on 31 December 2018 showed that with a population of 69.18 million citizens, the prevalence of tobacco use among people aged more than 15 years was 20.7%, and for younger ages of 13 to 15 years, was 15.0%. According to Thailand’s tobacco control plans from 2017 to 2021 [6], the smoking rate during the past 10 years has remained steady. However, the number of smokers was not reduced because of the low rate of smoking cessation, with an average of 30,000 to 40,000 people yearly. The low rate of smoking cessation is due to a lack of accessibility to smoking cessation services [6]. Furthermore, few smoking cessation services have been made available in Thailand. The insufficient amount of smoking cessation services and smokers’ lack of accessibility led to the creation of the community pharmacy smoking cessation service.

Various smoking cessation methods, techniques, and tools are available to help the smoker prepare, initiate, and maintain abstinence. The Fagerstrom nicotine-dependent test [7] is one of the tools used to determine the nicotine-dependent level of smokers to evaluate their dependence and assist them with appropriate smoking cessation methods and medication as needed. Additionally, the MPOWER strategy, promoted by the WHO, is also one of the approaches that healthcare professionals use in tobacco control, consisting of Monitoring tobacco use, Protecting people from tobacco smoke, Offering help to quit tobacco use, Warning about the dangers of tobacco, Enforcing bans on tobacco advertising and promotion, and Raising taxes on tobacco products [8]. In the WHO report on the global tobacco epidemic, 2019 [1], offering help to quit tobacco use is emphasized and encouraged to be acted upon. The letter “O-offering help” clarified that helping smokers through the quitting process has an impact when combined with other tobacco control strategies.

The pharmacist’s role in smoking cessation is to offer help to smokers. Community pharmacists play a crucial role in public health regarding smoking cessation services. Studies worldwide show that trained pharmacists assisting smoking cessation services are more efficient and lead to a sustained smoking cessation period [9,10,11,12]. They comprise professional healthcare personnel with a specialty in patient counseling and medication dispensing. The pharmacist can offer behavioral intervention using the 5A’s method to help smokers achieve their goals [13]. The 5A’s method—Ask, Advise, Assess, Assist and Arrange—is a popular method for smoking cessation. Furthermore, it can be used as a proactive tool to search for any smoker who is unaware of the smoking cessation services to receive the help needed. Despite there being efficient resources, like hospitals or healthcare units, they might be too unapproachable for some smokers. That is the reason why trained community pharmacists will constitute a suitable approach for effective smoking cessation services.

A community pharmacy is one of the public health services that has numerous advantages and already provides smoking cessation on a daily basis. Community pharmacies are easier to access in community areas, are not as crowded as hospitals, require fewer procedures, are not obliged by government operating hours, and provide a safe and friendly environment for patient counseling [14]. Community pharmacies can be easily spotted in almost every region in Thailand, and smokers can visit anytime without an appointment and receive the help required [15]. In community pharmacies, smoking cessation services are delivered by certified pharmacists providing intensive face-to-face behavioral smoking cessation counseling [10]. When the community pharmacist assesses whether the smoker needs medication to help with the smoking cessation process, the community pharmacist will be able to dispense the medication with utmost effectiveness and safety. Community pharmacists can also track the process of the smoker because they are conveniently located and friendly.

Thailand has an extended history of smoking cessation services [16,17,18]. Since 2004, the Thai Pharmacy Network for Tobacco Control (TPNTC) has promoted smoking cessation programs in community pharmacies in Thailand [16,17,18]. Although community pharmacists have helped clients quit smoking for more than 15 years, these services still do not cover all community pharmacies in Thailand due to many reasons such as lack of patient demand, lack of educational materials, lack of smoking cessation products, inadequate staffing, and being unable to follow-up [16,17,18]. Recently, the Foundation of Community Pharmacy (FCP), a nonprofit organization, has been dedicated to improving pharmacists’ role perceptions and creating engagement between community pharmacies and their local community. The FCP also provides essential materials and funds to any community pharmacies that need support for their proactive health programs, including smoking cessation activities [19]. Thus, this study aimed to evaluate the effectiveness of the community pharmacy smoking cessation services in Thailand.

## 2. Materials and Methods

This study collected data from a real-world practice of smoking cessation services in Thai community pharmacies of the FCP project from 1 July 2016 to 31 December 2017. This project covered 13 health regions, according to the National Health Security Office (NHSO), that covered all active community pharmacy smoking cessation services in Thailand. The study protocol was reviewed and approved by the human ethics committee of Khon Kaen University (Reference No. HE612074). All participants provided their consent to participate in the smoking cessation process and be included in this study.

### 2.1. The Foundation of Community Pharmacy (FCP)

The FCP is a nonprofit organization dedicated to improving pharmacist role perception and engagement between community pharmacies and the local community. The FCP database contains information on community pharmacies throughout Thailand. The FCP database constitutes an existing database of a developing project on community pharmacy smoking cessation services containing all recorded smoking cessation services and data on counseling, dispensing, physical measurements, such as exhaled carbon monoxide (CO) levels, and information about patient follow-ups.

### 2.2. Community Pharmacy Smoking Cessation Services

**Figure 1:**
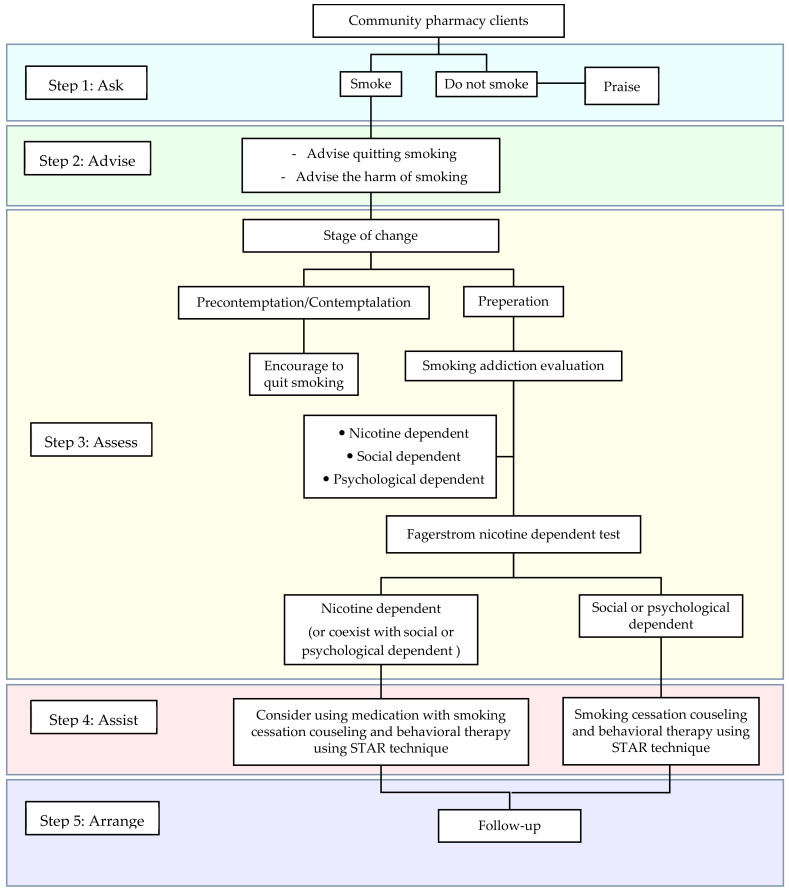
Pharmacist’s role in the community pharmacy smoking cessation service based on the 5A’s method.

The community pharmacies participating in this study offer smoking cessation services in local communities; some have offered these services for more than a decade [16,17,18]. The community pharmacists providing smoking cessation services were specifically trained for smoking cessation counseling. The major tools and strategies used for smoking cessation services are MPOWER, emphasized in O-offering help by patient counseling, and the 5A’s method described below and in Figure 1.

Ask (A1) involves pharmacists asking whether the participant is still smoking, has quit smoking, or has never smoked. Advice (A2) involves pharmacists advising and encouraging the smoker to terminate smoking ultimately. The pharmacist must use a calm manner, point out the harms of smoking, and explain the benefits of smoking cessation. Assess (A3) involves the pharmacist evaluating the willingness and readiness of the smoker to begin the smoking cessation process using a transtheoretical or Stage of Change model: precontemplation, contemplation, preparation, action, and maintenance. The pharmacists assessed whether the smoking addiction was psychological, social, or physical (e.g., nicotine dependence). The addiction to nicotine dependence was assessed using the Fagerstrom test. Assist (A4) involves the pharmacist offering appropriate help and patient counseling to help them terminate smoking entirely. Behavioral therapy and/or medication can be used in this process under close vigilance by a pharmacist. Because services can involve medication, a pharmacist is a key person to ensure the appropriate use of medication and its safety. The recommended tool is the STAR technique: Set a target quit date, Tell family members and others, Anticipate challenges, and Remove all tobacco-related products. Finally, Arrange (A5) involves pharmacists conducting a follow-up on the smoker’s progression. The smokers are praised when they successfully quit smoking, but when smokers cannot stop smoking, the pharmacist will consult the smoker individually to identify the problem or obstacle that prevents them from smoking cessation [13].

### 2.3. Participants and Data Collection

Eligibility criteria of participants included active smokers aged at least 18 participating in any community pharmacist-led smoking cessation service. Smokers were invited to join the service, including customers walking in the community pharmacy, and people participating in health-promoting events in community department stores or shopping malls which were proactive activities by community pharmacies arranged for their community, home visits, or service units regulated by a community hospital or primary care unit. Participants were excluded when they failed to provide complete required data for the analysis. The follow-up started when the participant was recruited and received smoking cessation services. The follow-up would continue as long as the participant can continuously stop smoking. The follow-up period differed depending on smoking status and the date that the participants entered the smoking cessation services. Nevertheless, the end of the follow-up period was the same date for every participant, which is the end date of the FCP project, 31 December 2017.

A retrospective data collection method was used. The data collection form was used to collect information about the community pharmacy smoking cessation services. Every community pharmacy in this study would report all the data to be recorded in the FCP database through the online website of the FCP project [19]. This data collection form included general information with the patient ID, age, sex, the community pharmacy where the patient was receiving services, provinces categorized in 13 health regions, and the source of patient (community pharmacy, health-promoting event, or service unit). In addition, the information about smoking behaviors was collected as the number of cigarettes smoked daily, type of addiction (psychological, social or physical), history of smoking cessation attempt, the motive to quit smoking, stage of change, and smoking cessation plan. The information of the smoking cessation process was also collected as the date of smoking cessation initiation, % peak expiratory flow rate (PEFR), exhaled CO level using PICO Smokerlyzer, and cigarette use.

### 2.4. Outcome Measurements

The study outcomes were the effectiveness of the community pharmacy smoking cessation services. The outcomes were the abstinence of smoking, which is defined by continuously stopping smoking for at least 30 consecutive days by self-report only, and self-report with an exhaled CO level of <7 ppm (when data were available), as well as the difference in the number of cigarettes smoked daily, exhaled CO level, PEFR using a Wright peak flow meter, and participants with PEFR <80%. Incomplete information on smoking cessation services or loss of follow-up was defined as being unable to quit smoking.

### 2.5. Statistical Analysis

Participant characteristics were analyzed using descriptive statistics; mean and standard deviations or median (interquartile range, IQR), as appropriate, were reported for continuous variables, and frequencies and percentages for categorical variables. The Shapiro–Wilk test was used to determine the normal distribution of a variable, and a nonparametric test was used when a variable was not normally distributed. The Wilcoxon Signed Rank test was used to analyze differences in the average number of cigarettes smoked daily, level of exhaled CO, and %PEFR before and after the process of smoking cessation services provided to the smokers. The McNemar test was used to analyze differences between participants with PEFR <80% before and after the process. All statistical analyses were conducted using STATA, Version 14, and the significance level was set at less than 0.05.

## 3. Results

**Figure 2:**
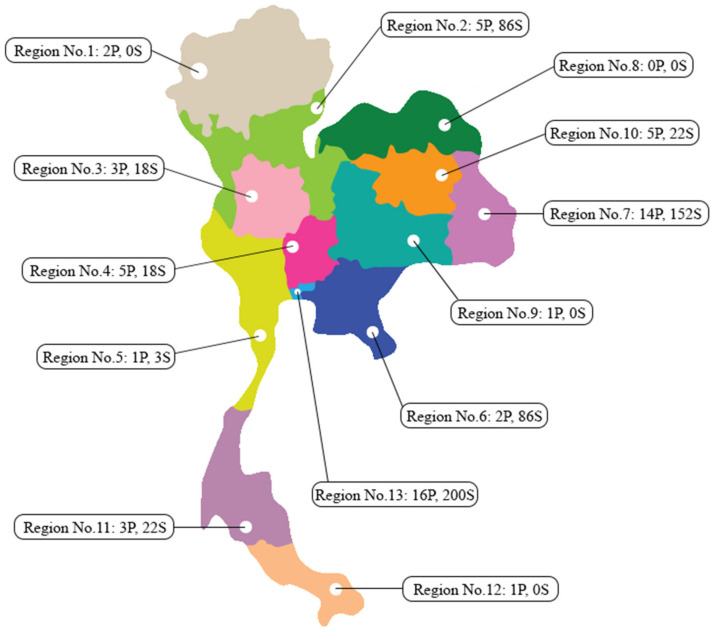
This figure shows the number of community pharmacies in this study covering 13 health regions, according to the National Health Security Office with the number of smokers who enter the community pharmacy smoking cessation program in each health region. For Example, Region No. 13: 16P, 200S is the health region number 13 had 16 participating community pharmacies, and had 200 smokers who experienced the community pharmacy smoking cessation service.

According to the NHSO, of 58 community pharmacies providing smoking cessation counseling services covering 13 health regions of Thailand, only 54 community pharmacies from 9 health regions were able to provide the complete data (Figure 2). Overall, 532 smokers volunteered to participate in this study. Most participants were men (92.0%), for which the previous average cigarettes smoked totaled 15.0 ± 9.3 daily before entering the project. As categorized in 13 health regions (regions 1 to 13), Region 13 (Bangkok) had the greatest number of community pharmacies and the most participants. Over 90.2% and 89.8% of the participants were psychologically and socially addicted to smoking, accordingly. The main reasons for deciding to quit smoking and participating in this service were for health (65.2%) and family members (44.9%). According to the Fagerstrom nicotine dependence test, the participants reported low (41.4%), moderate (40.1%), and high (18.5%) nicotine dependence (Table 1).

The results from 532 smokers entering the smoking cessation services, excluding 297 smokers with incomplete data, 235 smokers were able to provide complete information on smoking cessation counseling by community pharmacies. Of 532 smokers walking in the community pharmacy for smoking cessation services, 153 (28.8%) successfully stopped smoking for at least 30 consecutive days. Among community pharmacies where the device for measuring exhaled CO levels was available, all smokers (*n* = 52) providing a self-report of smoking cessation had exhaled CO levels <7 ppm. Comparing before and after receiving the counseling, the results indicated significantly reduced daily cigarette use from 15.3 ± 8.7 to 1.9 ± 3.8 cigarettes, *p*-value < 0.001. The participants’ exhaled CO levels were significantly reduced from 11.7 ± 5.9 ppm to 7.2 ± 4.4 ppm, *p*-value < 0.001. The %PEFR also significantly increased from 84.2 ± 19.4 to 89.5 ± 19.5, *p*-value < 0.001, and the number of smokers having PEFR <80% significantly decreased from 35.6 to 28.6%, *p*-value < 0.001 (Table 2). The two most frequently used medications in this study by 328 (61.6%) participants agreeing to take the medication as an additional aid besides smoking cessation counseling by the community pharmacist included nicotine gum (52.4%) and nortriptyline (26.2%).

## 4. Discussion

This study found that the community pharmacy smoking cessation services resulted in 28.8% of smokers quitting for at least 30 days, as identified by self-reporting. The self-reported smoking abstinence was shown to be reliable because it could be confirmed by the exhaled CO level. For every participant reporting smoking abstinence, the exhaled CO level was confirmed to be <7 ppm. In our study, only 15 of the community pharmacies were equipped with the PICO Smokerlyzer; the 52 participants’ self-reporting smoking abstinence with an exhaled CO level were recruited in a community pharmacy where the PICO Smokerlyzer was available. In addition, of the 52 participants who reported smoking abstinence, when tested with PICO, all were shown to have exhaled a CO level of <7 ppm. Due to this fact, the 153 people self-reporting only smoking abstinence might have resulted in an exhaled CO level of <7 ppm if they were to be measured by a PICO Smokerlyzer.

Although some of the smokers failed to quit smoking, participants had the number of cigarettes smoked daily, and the exhaled CO level significantly decreased with a significant increase of %PEFR after receiving the community pharmacy smoking cessation services. This aligned with the proportion of participants with %PEFR >80% which significantly increased after receiving smoking cessation services. Thus, our study suggested that even though not all smokers could stop smoking, smokers reduced the number of cigarettes used daily and increased their lung function, leading to better health.

The result showed that one of the main reasons why participants decided to quit smoking was health-related, indicating that smokers were fully aware of the negative effects of smoking in the first place. The objective evidence, the decreasing exhaled CO levels, was more relatable to the participants and made them feel like they were making progress and positively impacting their health. However, no evident markers or signs of illness or decline of health were observed, making it harder for the smoker to completely understand the dangers of smoking. Therefore, this study intended to show smokers the deteriorating effect of smoking on their bodies using %PEFR in addition to self-reporting. Participants were explained the definiteness of both measurements and their interpretations, so the results could be measured in as many visits as possible and presented to the participants. This revealed their progress and benefits of smoking cessation with actual measurable proof, encouragement, and pharmacist information. The participants were able to be more consistent on their mission and eventually quit smoking.

Compared with other recent studies related to smoking cessation in Portugal and Denmark [9,20], our study might appear to have had a lower smoking cessation success rate (28.8%) than the Portugal study that revealed a 43.7% success rate at one month. Furthermore, a study in Denmark had shown a higher success rate of 36.3% at the six-month follow-up period. That our study might appear to have a lower success rate might be due to two reasons. Firstly, the participants in the two studies expressed the willingness to quit smoking, while some smokers in our study did not have the willingness to quit but were motivated and encouraged to quit smoking by pharmacists. In our study, pharmacists asked smokers while they came for other services at the community pharmacy. In addition, the smoking cessation services were initially provided to participants at outreach activities, such as through health-promoting events and service units. Then the participants were referred to receive the full services at the community pharmacy. As a result, many smokers were encouraged and motivated to quit smoking using this process, which differed from the two studies. Secondly, our study method was flexible to suit the real-world situation where the community pharmacies were not obligated by the study protocol, while other study protocols might require strict collection of the data of smoking cessation services.

In addition, according to a study conducted in the U.S., over 39% of the participants were able to quit smoking in the two-month period. Nevertheless, the study outcome was the smoking abstinence of at least 7 consecutive days, which differed from our study that measured the smoking abstinence of at least 30 consecutive days. On the other hand, our study showed a higher success rate than some studies from Northern Ireland and Italy with similar outcomes [21,22]. However, those studies measured the outcome of smoking cessation for 24 weeks and 12 months, compared with one month in our study.

Community pharmacies are one of the major turning points in the pharmacist-assisted smoking cessation project. Because a community pharmacist can provide similar smoking cessation services with success rates equal to other healthcare professionals [23,24], they represent the right individuals for the job. The meta-analysis of the effectiveness of smoking cessation interventions in community pharmacies showed that community pharmacist interventions exhibited a higher abstinence rate than that of the control group [25]. The smoking cessation method was not easily achieved in a single visit. Hence, it requires multiple visits, patients, and consistency; both community pharmacies and pharmacists used appropriate approaches effectively for the process. However, some barriers exist to the smoking cessation process in Thailand, such as lack of knowledge and skills, lack of confidence, few smoking cessation products, and lack of public information media promoting community pharmacy smoking cessation service [16,17,18]. Community pharmacies are very common and conveniently accessed in Thailand and can reach out to the smoking community more easily compared with local hospitals or primary care units, which require more time, methods, and documents. Of over 58 community pharmacies in 13 health regions throughout Thailand participating in the study, 27.6% or the majority are in Region No.13, Bangkok, the capital of Thailand. 

Community pharmacies offering smoking cessation services are located throughout Thailand, resulting from an ongoing FCP project or the community pharmacy smoking cessation services that encourage proactive smoking cessation services by community pharmacies in Thailand. According to the Distribution of Community Pharmacies in the Context of Health Need in Thailand [15], over 16,053 community pharmacies exist in Thailand. Smoking cessation counseling is important, and community pharmacists are crucial for accurate and appropriate smoking cessation services. Furthermore, Thailand is going to support the health policy making a community pharmacy one part of universal coverage by promoting and developing the smoking cessation services. This policy makes the smoking cessation process much more convenient, effective, and approachable for both providers and smokers. By creating this health policy, community pharmacies participating in the FCP project are funded and provided with essential medication and tools to help with smoking cessation counseling. Furthermore, this project will reduce crowds and workloads for healthcare providers in hospitals.

A pharmacist is a crucial part of the effectiveness of the community pharmacy smoking cessation services. Because smoking is a major cause of many NCDs, and smoking cessation is a global NCD target [26]; the smoking cessation process should be handled with the utmost professionalism by healthcare providers. One of the tools used in the process is medication, and the pharmacist is the key person to assure that the patient will use the medication correctly and safely [27,28]. In MPOWER, the letter “O” stands for offering help to quit tobacco use. Both behavioral and pharmacotherapy interventions offered by the pharmacist in the process of counseling or product dispensing can double the rate of successful smoking cessation [29]. For instance, in a study about the pharmacist’s role in smoking cessation counseling, smokers currently or planning to quit using nonprescription NRT believed that receiving support and advice from a pharmacist would probably increase the smoker’s likelihood of smoking cessation [30]. Similarly to our study, smokers were motivated and encouraged by community pharmacists to quit smoking even though they did not plan to quit smoking. Moreover, some smokers in our study received smoking cessation medications and products (when needed) which were regulated and monitored by community pharmacists.

### Strengths and Limitations

Several strengths were identified in this study. Firstly, this study investigated elevated community pharmacy smoking cessation services in Thailand; therefore, this study involved community pharmacies nationwide. Community pharmacy smoking cessation services has been used to recruit and train community pharmacies throughout Thailand to provide approachable, friendly, and safe smoking cessation services. Therefore, the community pharmacists in this study were qualified and trained for smoking cessation counseling. Secondly, community pharmacy smoking cessation services eliminate the disadvantage of follow-up in the hospital. The hospital would appoint patients according to their routine medical schedule. The smoking cessation services in the hospital could not independently appoint patients on their own, but with a community pharmacy, patients could conveniently attend and receive the counseling at appropriate time interval [14]. Finally, our study reflected the real-world situation of the community pharmacy smoking cessation services.

However, this study encountered limitations. Firstly, due to the nature of the retrospective study design, missing data is possible, but we made maximum effort to ensure the quality of data. In this study, some participants were lost for follow-up; therefore, the complete information of smokers was not acquired. Secondly, the outcome measurements from exhaled CO levels using a PICO Smokerlyzer could not be determined in every community pharmacy. Lastly, the study lacked a control group, that is, the proper standard to evaluate intervention effectiveness. Despite the lack of a control group, this study revealed the health intervention situation of Thai community pharmacy smoking cessation services in the real world.

Due to the nationwide community pharmacies setting, this study could support policymakers to consider community pharmacy smoking cessation services to be part of the official national health program. Therefore, smokers could easily ask for smoking cessation services at a local community pharmacy.

## 5. Conclusions

This real-world study about community pharmacy smoking cessation services showed that by providing an adequate and smoking cessation process and using self-reports with an exhaled CO level to measure smoking cessation, the Thai community pharmacy could help smokers quit smoking. The community pharmacy smoking cessation services should be considered to be included in the national public healthcare policy to help smokers quit and proactively promote health for the community in which they serve.

## Figures and Tables

**Table 1:** Participant characteristics.

Characteristic	Frequency	Percent
Sex (*n* = 532)		
Male	495	93.0
Female	37	7.0
Age (years)		
Median (IQR)	43 (31, 55)	
Mean ± SD	42.4 ± 14.9	
Source (*n* = 532)		
Community pharmacy	386	72.6
Health-promoting event	95	17.9
Home visit	1	0.2
Service unit	25	4.7
Other	11	2.1
Not identified	14	2.6
Dependence condition (*n* = 532)		
Physical	307	57.7
Psychological	480	90.2
Social	478	89.8
Motivation to quit (*n* = 532)		
Family	239	44.9
Health	347	65.2
Social concern	60	11.3
Economic	60	11.3
Other	51	9.6
Nicotine dependence level (*n* = 524)		
Low dependence	217	41.4
Moderate dependence	210	40.1
High dependence	97	18.5
Stage of change (*n* = 512)		
Precontemplation	9	1.8
Contemplation	101	19.7
Preparation	357	69.7
Not identified	45	8.8
History of smoking cessation attempt	361	67.9
Number of initial cigarettes smoked daily		
Median (IQR)	13.5 (10.0, 20.0)	
Mean ± SD	15.0 ± 9.3	
Quit smoking plan (*n* = 532)		
No medication	204	38.3
Use medication	328	61.6
Medication used for smoking cessation (*n* = 328)		
Nicotine gum	172	52.4
Nortriptyline (10, 25 mg)	86	26.2
Bupropion	5	1.5
Nicotine patch	3	0.9
Other (gargling with sodium nitrate, Vernonia cinerea lozenge, Vernonia cinerea tea)	62	18.9

**Table 2:** Outcomes of smoking cessation services by the community pharmacy smoking cessation services.

Outcome of Smoking Cessation Services	*n*	Before	After	*p*-Value
Smoking abstinence (≥30 days)				
Self-report	235	0	153 (28.8%) (95% CI: 24.9–32.6%)	-
Self-report and exhaled CO level (<7 ppm)	235	0	52 (9.8%) (95% CI: 7.3–12.3%)	-
Number of cigarettes smoked daily				
Median (IQR)	235	15.0 (10.0, 20.0)	0 (0.0, 3.0)	<0.001 *
Minimum, maximum	235	1, 40	0, 20	
Level of exhaled CO (ppm)				
Median (IQR)	137	11.0 (7.0, 16.0)	6.0 (4.0, 10.0)	<0.001 *
Minimum, maximum	137	1, 28	1, 18	
% PEFR				
Median (IQR)	227	86 (74, 95)	89 (77.9, 102)	<0.001 *
Minimum, maximum	227	26, 157	34.5, 157	
Participants with PEFR less than 80% (percent)	227	83 (35.6)	65 (28.6)	<0.001 **

Note: * Wilcoxon Signed Rank test; ** McNemar’s test; PEFR, peak expiratory flow rate; IQR, interquartile range; CO, carbon monoxide; ppm, parts per million; CI, confidence interval.

## Data Availability

The data presented in this study are available on reasonable request from the corresponding author.
